# Magnetic nanoparticles in theranostics of malignant melanoma

**DOI:** 10.1186/s13550-021-00868-6

**Published:** 2021-12-14

**Authors:** Maxim Shevtsov, Susanne Kaesler, Christian Posch, Gabriele Multhoff, Tilo Biedermann

**Affiliations:** 1 Central Institute for Translational Cancer Research (TranslaTUM), Radiation Immuno-Oncology Group, Klinikum rechts der Isar, School of Medicine, Technical University Munich (TUM), Einstein Str. 25, 81675 Munich, Germany; 2 Laboratory of Biomedical Cell Technologies, Far Eastern Federal University, Primorsky Krai, 690091 Vladivostok, Russia; 3 Personalized Medicine Centre, Almazov National Medical Research Centre, 2 Akkuratova Str, Saint Petersburg, Russian Federation 197341; 4 Department of Dermatology and Allergology, Klinikum rechts der Isar, School of Medicine, Technical University Munich (TUM), Biedersteinerstrasse 29, 80802 Munich, Germany; 5 Department of Radiation Oncology, Klinikum rechts der Isar, School of Medicine, Technical University Munich (TUM), Ismaninger Str. 22, 81675 Munich, Germany

**Keywords:** Melanoma, Magnetic nanoparticles, SPIONs, Theranostics

## Abstract

Malignant melanoma is an aggressive tumor with a tendency to metastasize early and with an increasing incidence worldwide. Although in early stage, melanoma is well treatable by excision, the chances of cure and thus the survival rate decrease dramatically after metastatic spread. Conventional treatment options for advanced disease include surgical resection of metastases, chemotherapy, radiation, targeted therapy and immunotherapy. Today, targeted kinase inhibitors and immune checkpoint blockers have for the most part replaced less effective chemotherapies. Magnetic nanoparticles as novel agents for theranostic purposes have great potential in the treatment of metastatic melanoma. In the present review, we provide a brief overview of treatment options for malignant melanoma with different magnetic nanocarriers for theranostics. We also discuss current efforts of designing magnetic particles for combined, multimodal therapies (e.g., chemotherapy, immunotherapy) for malignant melanoma.

## Introduction

Cutaneous melanoma (CM) is a tumor of the skin accounting only for approximately 4% of all skin tumors, but it causes most skin-cancer-related deaths [1]. The incidence of CM is growing and has drastically increased especially in the last 50 years when compared to other malignant tumors [2]. For 2021, 5.8% more newly diagnosed cases and 4.8% more deaths are expected in the USA*.* CM is a highly aggressive tumor with a propensity to metastasize early. The relative 5-year survival rate based on the time of initial diagnosis is 99% for localized CM, but decreases to 66% and 27% after regional spread and distant metastasis, respectively (American Cancer Society. *Cancer Facts & Figs. 2021. Atlanta: American Cancer Society; 2021*).

Melanoma develops from melanocytes which are neural crest-derived pigmented cells mainly found in the dermoepidermal junction and hair follicle [3]. Several factors can contribute to the transformation of melanocytes, but exposure to ultraviolet (UV) radiation is thought to be the predominant environmental risk factor [4]. This includes recurrent sunburns and frequent and extensive sunbathing [5] by indoor tanning, in particular in younger age-groups (< 30 years) [6]. Consistent with this, CM compared to other tumor entities is characterized by a high mutational burden with typical UV signatures [7, 8]. Other risk factors include a fair skin phenotype (fair complexion, blond or red hair, blue eyes, tendency to freckle) [9], the number and type of naevi [10, 11], and a personal or family history of melanoma [12–14].

For a long period of time, only few therapeutic options, including surgery, chemo- and radiotherapies, the development of immune checkpoint inhibitors and targeted therapies have significantly improved the outcome of CM. Yet, up to 50% of all metastatic patients do not benefit from modern melanoma therapy due to primary or secondary resistance. The current strategy is to overcome these problems with combined therapies that facilitate known and new molecular melanoma vulnerabilities. In addition to new therapeutic approaches, this requires a profound knowledge of the regulation of the immune system such as mechanisms that induce tolerance and suppression or activate effector cells as well as of key signaling pathways in melanoma biology [15–22].

In recent decades, nanoparticles (NPs) have emerged as a new theranostic modality for the treatment of melanoma patients [23]. Employment of nanotechnologies has greatly improved the early diagnosis and the therapy of cancer by providing novel strategies for a targeted delivery of anti-tumor agents (e.g., drugs, anti-proliferative proteins, etc.), and genes to the site of tumor [24–26]. Nanoscale agents can originate from inorganic (e.g., iron, superparamagnetic iron oxide, gold, mesoporous silica, graphene and carbon, etc.) and organic nanomaterials (e.g., lipids, proteins, silica, carbohydrates, etc.) of various formulations and shapes (e.g., spheres, nanotubes, quantum dots) [27–40]. Among the proposed nanocarriers, metal-based NPs, particularly magnetic nanoparticles (MNPs), gained much attention due to their beneficial physicochemical properties.

Among other properties of MNPs, excellent magnetic contrast-enhancing properties, biodegradability and biocompatibility gained specific interest in clinical oncology [41, 42]. Thus, magnetic particles could significantly improve the magnetic resonance contrast enhancement of the tumors when being applied as T_2_ contrast agents [43, 44]. Additionally, MNPs could be used either for heating of the tumors in an alternating electromagnetic field (AMF) or for a targeted delivery of anti-tumor agents [45, 46]. The clinical relevance of MNPs is further supported by the fact that several iron oxide nanoparticle formulations have been approved by the Food and Drug Administration (FDA) as MR contrast agents, including Feridex IV® for detection of liver lesions and Combidex® for visualization of lymph nodes metastasis [47, 48]. Recent advances in the physicochemical formulations of NPs including surface modifications such as binding of various tumor-homing ligands (e.g., antibodies, Fab-fragments, peptides, etc.) have significantly broadened the potential of MNPs application in translational and clinical dermato-oncology.

In the current review, the application of NPs for diagnosis and therapy of malignant melanoma is discussed with a special focus on translational studies. Additionally, we describe currently applied combined therapeutic approaches of MNPs together with other treatment modalities.

## Current treatment strategies of malignant melanoma

Depending on a histopathological combination of tumor thickness with or without ulceration, and the presence of local, lymph node or distant metastasis (TNM system), the American Joint Committee on Cancer (AJCC) classified melanoma in five different stages [49], which are important for treatment decisions. The prognosis worsens with increasing stage, it starts with stage 0, the melanoma in situ, which is restricted to the epidermis without any indication of invasion, and ends with stage 4, advanced malignant melanoma, which has already spread to distant parts of the body [49, 50]. While early disease is limited to the epidermis (melanoma in situ, “Tis”) and most melanomas with a tumor thickness of less than 1 mm can be cured surgically, metastatic disease requires multidisciplinary treatment approaches.

For a long time, surgery, chemotherapy and radiation were the only available therapeutic options, but in most cases response rates were low and patients with advanced tumor stages had a short life expectancy [51]. The cytostatic drug dacarbazine, approved in 1974 by the FDA (Table 1), has long been used for systemic treatment of metastatic melanoma, but with low response rates and without improvements in overall survival [52, 53]. Immunotherapies with interferon (IFN)α-2b and interleukin (IL)-2 also failed to result in high response rates [54, 55]. However, anti-tumor effects have been reported for both cytokines [56, 57], and both cytokines are presently still used in combination with other treatment modalities in clinical trials (Clinical trials.gov). An improved pathomechanistic understanding has led to a paradigm shift in the last 10 years, and treatment options for malignant melanoma dramatically changed due to the development of new innovative systemic and local therapies. The use of immune checkpoint inhibitors on the one hand and the targeted treatment of tumor-specific genetic alterations with kinase inhibitors on the other hand significantly contributed to this success.

**Table 1:** FDA-approved drugs for the treatment of metastatic melanoma

Drug	Active ingredient	Mechanism	FDA approval
Dacarbazine	Imidazole carboxamide, alkylating agent	Cytostatic; blocks cell division by methylation of DNA component	1975
Interferon a-2b	Cytokine	Adjuvant therapy, immunostimulant	1995
Interleukin -2 (IL-2)	Cytokine	Adjuvant therapy, immunostimulant	1998
Vemurafenib	Small molecule	Protein kinase inhibitor targeting mutated BRAF	2011
Ipilimumab	Antibody, checkpoint inhibitor	Blocks the immune inhibitory receptor CTLA-4	2011
PEG-IFN alpha-2b	Cytokine	Adjuvant therapy with reduced clearance of agent	2011
Dabrafenib	Small molecule	Protein kinase inhibitor targeting mutated BRAF	2013
Trametinib	Small molecule	Protein kinase inhibitor targeting MEK1/2	2013
Dabrafenib + Trametinib	Small molecules	Protein kinase inhibitor targeting mutant BRAF and MEK1/2, respectively	2014
Nivolumab	Antibody, checkpoint inhibitor	Blocks the immune inhibitory receptor PD-1	2014
Pembrolizumab	Antibody, checkpoint inhibitor	Blocks the immune inhibitory receptor PD-1	2014
Talimogene laherparepvec	Modified herpes simplex virus	Induction of cell lysis	2014
Vemurafenib + Cobimetinib	Small molecules	Protein kinase inhibitor targeting mutant BRAF and MEK1/2, respectively	2015
Nivolumab + Ipilimumab	Antibodies, checkpoint inhibitors	Block the immune inhibitory receptors PD-1 and CTLA-4, respectively	2015
Encorafenib + Binimetinib	Small molecules	Protein kinase inhibitor targeting mutant BRAF and MEK1/2, respectively	2018
Atezolimab + Vemurafenib + Cobimetinib	Antibody and small molecules	Immune checkpoint inhibitor against PD-L1 and protein kinase inhibitors	2020

Table with chronological listing of drugs approved by the FDA for the treatment of malignant melanoma

### Immune checkpoint inhibitor therapies

Immune checkpoints are important regulatory elements of the immune system. As gatekeepers, they prevent overshooting and autoreactive immune responses by a mechanism dependent on a ligand-induced signaling. In 2011, the FDA approved the first checkpoint inhibitor (ICI) ipilimumab for the treatment of metastatic melanoma (Table 1). Ipilimumab is a human monoclonal antibody that binds to the cytotoxic T-lymphocyte-associated protein 4 (CTLA-4), a surface molecule expressed on T cells after T cell receptor (TCR) engagement [58]. Checkpoint inhibitor binding to CTLA-4 prevents a negative feedback loop and maintains T cells in an activated, proliferating state. Although the response rate to anti-CTLA-4 monotherapy was generally low, in case of a response it was long-lasting and significantly increased overall survival [59, 60]. The disadvantage of this non-specific treatment with anti-CTLA-4 is several immune-related adverse events (irAEs) [59, 61]. Approvals for other ICIs such as programmed cell death protein 1 (PD-1) blocking antibodies nivolumab and pembrolizumab followed in 2014 (Table 1). Compared to CTLA-4, PD-1 is also expressed on activated T cells and NK cells, at a lower density in primary lymphoid tissues, but in the periphery [62]. Compared to ipilimumab, the PD-1 inhibitors reached even higher overall response rates of 30–40% [63], with less irAEs and longer relapse-free and overall survival rates [64, 65]. Currently, for patients with unresectable metastatic melanoma anti-PD-1 blockage alone or in combination with CTLA-4 blockage is recommended and approved as a first-line treatment [50]. Unfortunately, a large proportion of patients still does not benefit from ICIs because they either initially do not respond or develop resistances during the course of treatment [64, 66, 67]. The reasons for this are complex and include immunosuppressive factors of the tumor microenvironment, immune editing, lack of neoantigens, loss of antigen presentation, heterogeneity of the melanoma tumor cells and an impaired function of tumor-infiltrating T and NK cells [64, 68–75]. A summary of FDA-approved therapy options for metastatic melanoma is shown in Table 1.

### Targeted therapies

With a high mutational burden [7], CM provides a wide-ranging landscape of genomic alterations. Mutations in the mitogen-activated protein kinase (MAPK) signaling pathway are among the most common genetic alterations in CM. MAPK signaling cascades are evolutionarily conserved, complex pathways that transfer extracellular signals to intracellular responses, thereby controlling many cellular processes, including proliferation, differentiation, migration and apoptosis [76]. Three MAPK cascades—the extracellular signal-regulated kinases (ERK), p38 MAPK and c-Jun-N-terminal kinase (JNK)—have been intensively studied in mammalian cells, in which the binding of an extracellular signal to a membrane-bound receptor activates a multistep phosphorylation pathway. In the ERK1/2 pathway, this is the RAS-RAF-MEK-ERK cascade, initially activated by the binding of a mitogenic factor to its receptor. Dysregulation of the ERK1/2 pathway mainly due to an activation of genetic alterations is most often involved in oncogenesis [77] and is associated with an increase in the growth and proliferation of tumor cells [78, 79]. In melanoma, this is of particular interest, as about 50% of the melanomas show mutations in BRAF (a member of the RAF family), about 25% show mutations in NRAS (a member of the Ras family) [80, 81] and around 15% have mutations in NF1 (neurofibromin), a tumor suppressor that negatively regulates Ras. Based on these most commonly mutated genes, The Cancer Genome Atlas (TCGA) Network established a genomic classification of melanoma into four subtypes: mutant BRAF, mutant NRAS, mutant NF1 and triple wildtype [82]. About 90% of BRAF mutations in CM involve amino acid 600 with an exchange of valine to glutamic acid (BRAf^V600^^E^), resulting in an enhanced, Ras-independent activation of MEK [83] and an increased proliferation of the affected cells. With Vemurafenib, a sulfonamide that selectively inhibits the BRAF^V600E^ kinase, the first BRAF inhibitor for unresectable or metastatic melanoma was approved in 2011 [84–86]. Dabrafenib, a second-line BRAF^V600E^-specific kinase inhibitor followed shortly afterward [87, 88] (Table 1). Although the treatment initially showed promising therapeutic effects (even complete remissions), relapses occurred within 8–12 months due to (mainly secondary) resistance mechanisms with alterations leading to reactivation of the MAPK pathway [84, 87, 89]. Furthermore, about 10% of patients did not respond to the drug at all due to an intrinsic, primary resistance [90]. To counteract this effect, a combination of inhibitors targeting BRAF and MEK, the downstream kinase of BRAF, was tested. Trametinib, a MEK inhibitor licensed in 2013 for monotherapy, was approved in 2014 for the combined treatment with dabrafenib [91], others followed in 2015 (vemurafenib and cobimetinib) [92] and in 2018 (encorafenib and binimetinib) [93] (Table 1).

### Combination of checkpoint inhibitor and targeted therapy

Interestingly, BRAF inhibitors also seem to have immunomodulatory properties by impacting melanoma antigen presentation [94], tumor-infiltrating T cells [95] and cytokines indicative for immune response [96], suggesting that the tumor microenvironment is less immunosuppressive under this treatment regimen. Based on this, ICIs have been combined with targeted therapies. The combination with atezolimab (anti-PD-1 antibody) plus cobimetinib and vemurafenib showed a significant improvement compared to targeted therapy alone and was approved for the treatment of BRAF^V600^^E^-positive patients with metastatic melanoma by the FDA in 2020 (Table 1). Today, it still remains unclear which patients benefit most from a triple therapy consisting of BRAF plus MEK plus PD-1 inhibition and whether this treatment is superior to a combined PD-1 and CTLA-4 checkpoint blockade. Significant treatment-related side effects also limit the use of combined targeted and immunotherapy.

### Oncolytic virus therapy

In 2015, the FDA approved a therapy with modified herpes simplex virus type I, talimogene laherparepvec (T-VEC), for melanoma patients with locally advanced disease. T-VEC lacks a virulence gene, an immunogenicity gene but contains a gene to express the human granulocyte macrophage colony-stimulating factor (GM-CSF) [97] (Table 1). T-VEC is injected directly into metastatic lesions, where the virus selectively replicates in tumor cells, causing them to lyse, while healthy tissue remains unaffected. A systemic immune response is also induced by tumor cell lysis [97]. The treatment resulted in improved durable responses, objective response rates and progression-free survival in a randomized phase III clinical trial for patients with locally advanced melanoma [98, 99], although overall survival was not improved. In most cases, only injected lesions responded to therapy. Thus, T-VEC has to be considered as a local treatment.

**Fig. 1:**
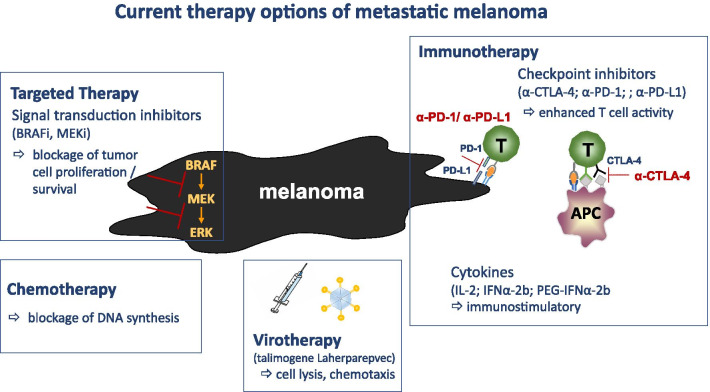
Schematic overview of currently applied therapies for the treatment of malignant melanoma. Abbreviations: T: T cells; APC: antigen-presenting cell; CTLA-4: cytotoxic T-lymphocyte-associated protein-4; PD-1: programmed cell death protein 1; PD-L1: PD ligand 1; i: inhibitor

**Fig. 2:**
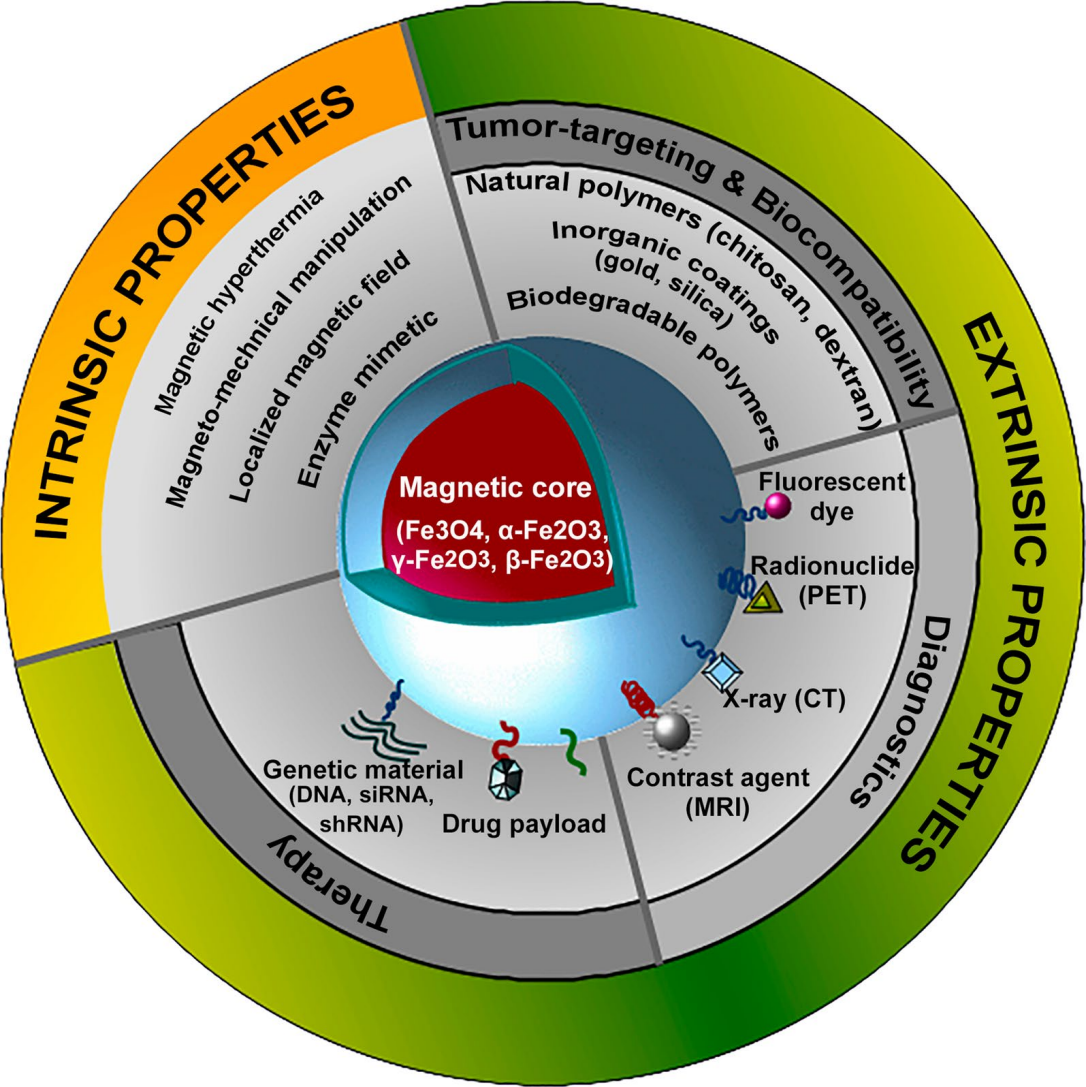
Schematic representation of magnetic nanoparticle and its application in melanoma theranostics

Considering the increasing incidence and aggressiveness of melanoma, prevention and early diagnosis are key for combating melanoma. Nevertheless, approximately 10% of patients already have advanced, metastatic melanoma at first diagnosis. Furthermore, a large proportion of affected patients currently do not benefit from the available therapies and it remains challenging to develop more effective approaches. Figure 1 provides a schematic overview of presently FDA-approved treatment options including targeted therapy, chemotherapy, virotherapy and immunotherapy for patients with malignant melanoma. Significant progress in nanotechnology has already been made with the emerging of new biomedical nanoplatforms, particularly in the development of MNPs that can be applied for theranostic purposes in melanoma. Currently, the NIH database of the U.S. National library of Medicine (ClinicalTrials.gov) lists only 10 clinical trials worldwide using NPs for the treatment and/or diagnostics of malignant melanoma (Table 2) [100–104]. Due to their unique intrinsic physicochemical properties, MNPs can be used for imaging and therapy after coating with dyes, radionuclides, antibodies, drugs, etc.; drug release and positioning of the MNPs can be controlled in a localized magnetic field; different modes of cell death (e.g., ferroptosis) and thermotherapy can be induced in an alternating electromagnetic field (Fig. 2). Tailoring of extrinsic properties of NPs by introducing biocompatible and biodegradable coatings, surface modifications with various bioligands, incorporation of various molecules with diagnostic and therapeutic properties can increase their tumor-targeting (and thus decrease off-target side effects) and theranostic potential.

**Table 2:** Clinical trials using nanoparticles with melanoma patients

Clinical trial no.	Phase	Patients	Study design	Status	Purpose	Refs
NCT00626405	II	Unresectable stage IV melanoma	Bevacizumab + Temozolomide vs Bevacizumab + paclitaxel albumin-stabilized nanoparticle formulation + carboplatin	Completed	Treatment	[100]
NCT00081042	II	Unresectable stage IV melanoma	Paclitaxel albumin-stabilized nanoparticle formulation; previously chemotherapy vs chemotherapy-naive	Completed	Treatment	[101]
NCT00738361	II	Unresectable metastatic uveal melanoma	Paclitaxel albumin-stabilized nanoparticle formulation; single group	Completed	Treatment	
NCT00404235	II	Unresectable stage IV melanoma	Paclitaxel albumin-stabilized nanoparticle formulation; chemotherapy-naïve; single group	Completed	Treatment	[100]
NCT02158520	II	Unresectable stage IV melanoma	Bevacizumab + paclitaxel albumin-stabilized nanoparticle formulation vs ipilimumab	Completed	Treatment	[103]
NCT01300533	I	Advanced or metastatic cancer including melanoma	BIND-014: targeted docetaxel polymeric nanoparticle; single group	Completed	Treatment	[104]
NCT02668536	I	Melanoma and UV ray damaged skin	Standard sunscreen vs sunscreen based on bioadhesive nanoparticles	Completed	Prevention	[105]
NCT04899908	II	Cancer with brain metastasis including melanoma	Stereotactic radiation with vs without AGuIX gadolinium-based nanoparticles	Recruiting	Treatment	
NCT03739931	I	Advanced malignancies including melanoma	Lipid nanoparticle encapsulating mRNAs with vs without durvalumab	Recruiting	Treatment	
NCT02106598	II	Head and neck melanoma	Silica nanoparticles with fluorescent cRGDY-PEG-Cy5.5-C dots for real-time image-guided intraoperative mapping of nodal metastases	Recruiting	Diagnostic	

Completed and recruiting clinical studies currently listed in the NIH database of the U.S. National Library of Medicine(https://clinicaltrials.gov)

## Nanoparticles in melanoma theranostics

### Basic principles of nanomaterials

Organic and inorganic nanomaterials can improve diagnosis and therapy of melanomas. Liposomes with a high biocompatibility can be utilized as drug vehicles for a targeted delivery of anti-tumor agents, while sparing normal tissues [105]. Changing the composition, size, shape and load of NPs can alter tissue specificity, pharmacokinetics and tumor-homing capacity of NPs [23]. Inorganic (e.g., metal- or non-metal-based) nanomaterials harbor theranostic potential: On the one hand, they can be used as vehicles for drug delivery; on the other hand, they improve the monitoring of response of a tumor toward a drug by utilizing its imaging properties in MR [43, 44, 47]. Coating of NPs with biological materials is able to improve biocompatibility of metal-based nanomaterials in vivo. Functionalization of NPs with tumor-specific antibodies, proteins, peptides, enzymes [34], dyes, radionuclides, etc., enhances their efficacy and tumor-specific targeting. Due to their conductivity properties, metal-based nanomaterials are able to induce local hyperthermia at the site of the tumor [46] (Table 3).

**Table 3:** Magnetic nanoparticles in melanoma theranostics

Nanoparticles		Method	Mode of action	Refs
Intrinsic properties	Magnetic core	Magnetic hyperthermia	Induction of apoptosis	[106]
			Impaired cell differentiation and proliferation due to protein alteration	[107–110]
			Irreversible cell damage	[68, 69]
			Immunostimulation	[45, 116, 117]
			Photothermal therapy	[121–124]
		Magneto-mechanical effects	Cell damage, apoptosis induction	[128–130]
			Damage of neovasculature	[131]
			Magnetoporation, magnetolysis	[132, 133]
		Localized magnetic field	Drug release	[134–138, 141–144]
		Nanozyme	Induction of ferroptosis	[145–150]
Extrinsic properties	Coating with attachments	Release of chemotherapeutic agents	Cytotoxicity, apoptosis	[152–158]
		Immunoadjuvants	Immunostimulation	[159, 165]
		Tumor-targeting molecules		[143, 169]
		Labels for imaging	Diagnostics	[172—175]

### Magnetic hyperthermia (MH)

Magnetic NPs typically consist of an iron oxide core (including magnetite (Fe_3_O_4_), hematite (α-Fe_2_O_3_) and maghemite (γ-Fe_2_O_3_ and β-Fe_2_O_3_)) coated with biocompatible and biodegradable polymers (e.g., dextran, polyethylene glycol (PEG), polylactic-co-glycolic acid (PLGA), etc.). Due to their magnetic properties, NPs can be employed for thermotherapy via an increase in the temperature inside the tumor (ranging from 41 to 46 °C) and an induction of apoptotic signaling cascades [106]. Additionally, the rise in temperature alters the enzymatic activity and structure of numerous proteins and affects the synthesis of the nucleic acids that in turn impairs cell differentiation and proliferation [107–110]. A further increase in the temperature (above 50 °C), that is employed for thermal ablation, results in irreversible cellular damage due to coagulation and necrosis [68, 69].

When exposed to an external alternating magnetic field, magnetic particles generate the heat via the mechanisms of hysteresis loss and Brownian and Néel relaxation [111, 112]. Indeed, several preclinical studies reported the efficacy of the MH in melanoma treatment [113–115]. Thus, highly focalized thermotherapy in the B16F10 melanoma model in C57/Bl6 mice inhibited the tumor growth by 70% as compared to a sham-treated control group [115]. Intriguingly, in another study MH resulted in a decrease in transforming growth factor (TGF)-β(1) protein expression that also might have an impact on the tumor progression [114].

One of the future efforts in the application of MH in melanoma treatment could be based on the immunostimulatory effects of hyperthermia. As shown by Duval et al., modest magnetic hyperthermia of B16 melanoma cells induced the expression of various immunogenic genes including heat shock protein (Hsp)70, CXCR3, and innate immune activators Toll-like receptor (TLR)3, TLR4 [116]. Further in vivo studies demonstrated that localized MH strongly correlated with the expression of Hsp70 in the tumor and the influx of activated cytotoxic CD8^+^ T lymphocytes [45, 117]. Presumably, combination of MH with other immunotherapeutic approaches might have a synergistic therapeutic effect [118, 119]. Hoopes et al. reported application of MH (43 °C/60 min) of intratumorally delivered immunoadjuvant plant-based virus-like nanoparticle VLP (4 × 200 µg) and magnetic NPs (2 × 7.5 mg/g tumor) combined with hypofractionated radiotherapy in the canine oral melanoma patients. The authors demonstrated an increased immune cell infiltration into the tumor and extended tumor control intervals [120].

Further modifications of magnetic NP formulations can increase their theranostic properties. Thus, hybrid gold ferric oxide NPs enable the magnetic targeting of NPs to the tumor site for subsequent photothermal therapy [121–123]. Subsequent application of magnetically targeted nano-photothermal therapy based on Fe3O4@Au NPs decreased tumor progression in a preclinical melanoma model [124]. Furthermore, in the recent report by Zhang et al. it was demonstrated that NPs could be used as a platform for the multimodal theranostics in melanoma [125]. Thus, MSN(Mn)-ICG/DTIC NPs (that incorporated dacarbazine (DTIC), indocyanine green (ICG), mesoporous silica NPs (MSN(Mn))) achieved a significant anti-tumor chemo-photothermal effect [125].

### Magneto-mechanical manipulation

Therapeutic approaches based on magneto-mechanical effect of particles are a growing field in the treatment of tumors [126, 127]. Upon application of the external magnetic field, internalized NPs align themselves to the plane of the rotating magnetic field, creating a strong mechanical force which damages tumor cells and induces apoptosis [128]. Oscillation of the NPs under a low-frequency magnetic field can result in the mechanical stretching of the cytoskeleton and an impairment of the ion channel activity [129]. Furthermore, after lysosomal targeting (via antibodies targeting the lysosomal protein marker LAMP1), rotating NPs damage lysosomal membranes and thus induce apoptotic cell death [130].

**Fig. 3:**
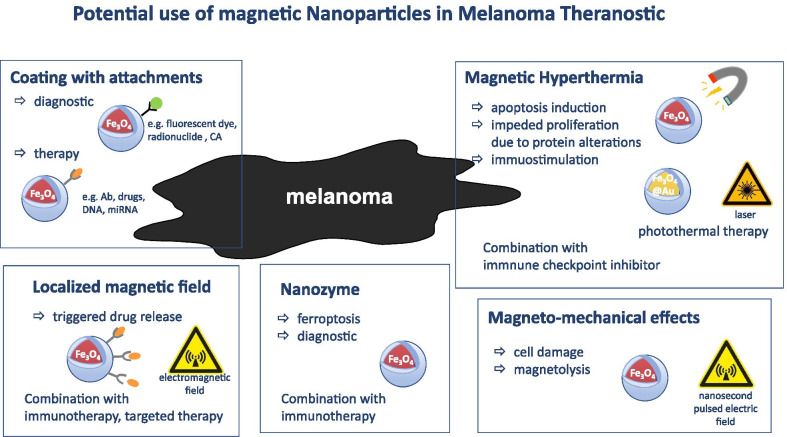
Schematic overview of the potential use of nanoparticles alone or in combination with approved treatment options in malignant melanoma, exemplified for nanoparticles with Fe_3_O_4_ core. Abbreviations: CA: contrast agent; Ab: antibody

One of the promising approaches for the treatment of melanoma could be based on nanosecond pulsed electric fields (nsPEF) that have thus far been evaluated in vitro and in superficial malignancies, in vivo. Previously, Bardet et al. demonstrated for the first time that a single 10 ns, high-voltage electric pulse (35–45 kV/cm), collapses the perfusion of the neovasculature and alters the diameter of capillaries and larger vessels in normal tissue [131]. Furthermore, weak magnetic fields (40–75 mT) applied on tumor cells containing polymer-coated multi-walled carbon nanotubes induce magnetoporation of tumor cell membranes and tumor cell death by magnetolysis [132, 133] (Fig. 3).

Although magneto-mechanical approaches demonstrated a therapeutic potency in vitro, only a few in vivo studies were reported with highly heterogenous magnetic nanocarriers. Further preclinical studies in clinically relevant melanoma models are mandatory to decipher the mechanisms underlying the observed therapeutic effect (particularly taking into consideration the recent advances in mechano-transduction pathways).

### Localized magnetic field for drug release

Application of drug-eluting beads triggered by the external alternating magnetic field demonstrated promising results in various preclinical models [134–138]. Indeed, encapsulation of anti-melanoma drugs in triggerable magnetic NPs can beneficially modify biodistribution and pharmacokinetics of therapeutic agents (thus increasing on-site drug concentration and reducing off-target side effects) and trigger the release of the chemotherapeutic compound by electromagnetic field in the required time period [139, 140] (Fig. 3). Additionally, magnetic NPs as shown by numerous studies can induce vasodilatation that increases blood circulation and thereby enhances chemotherapeutic drug delivery. Furthermore, employment of the magnetoresponsive particles facilitated the doxorubicin release and its efficient distribution inside the tumor tissue upon application of low-frequency (Lf) electromagnetic-induced magnetophoresis [135] (Fig. 3). In another study, a radical initiator (AIPH) loaded into porous hollow iron oxide nanoparticles (PHIONs) under AMF resulted in production of oxygen-independent alkyl radicals with significant therapeutic potency [141]. Colloidally stable core–shell cobalt ferrite@barium titanate (CFO@BTO) ME NPs were shown to release doxorubicin and methotrexate upon application of the magnetic field (5 mT) inhibiting the growth of human malignant melanoma cells HT144 [142]. In another study, cell-penetrating peptides (CPP) and tumor necrosis factor (TNF)-α (CTNF-α)-anchored exosomes coupled to superparamagnetic iron oxide NPs (CTNF-α-exosome-SPIONs) showed a membrane-targeting anticancer activity in a melanoma model when external magnetic fields were applied [143]. In the recently published work of García-Hevia et al., the authors developed a nanoplatform based on magnetic lipid nanocomposite vehicles (mLNVs) loaded with doxorubicin that demonstrated a therapeutic potency in B16 mouse melanoma models [144]. Presumably, a combination of a triggered release of anti-melanoma drugs with other therapies (e.g., radiotherapy, immunotherapy, targeted therapy, etc.) could further increase the therapeutic potential of the magnetic particles (Fig. 3).

### Enzyme mimetic for melanoma therapy

In 2007, it was demonstrated for the first time that metal NPs, particularly magnetite (Fe_3_O_4_) particles, possess an intrinsic enzyme mimetic activity similar to that of peroxidase [145]. This discovery triggered the development of a new class of catalytic agents that were termed “*nanozyme*,” thus distinguishing nanomaterials with intrinsic enzyme properties from other particles with externally immobilized enzymes [146] (Fig. 3). Up to date, more than 300 nanomaterials are described with enzyme-mimicking activity [147]. Currently, nanozyme has demonstrated to mimic activities of enzymes belonging to the oxidoreductase family (i.e., catalase and peroxidase) and therefore can also be applied for cancer theranostics [145, 148]. The developed nanoparticle-based sensor platform successfully identified circulating tumor cells in melanoma by catalyzing the oxidation of TMB (3,3',5,5'-tetramethylbenzidine) into a blue-colored product [25]. Subsequent studies reported that magnetite NPs catalyzing decomposition of hydrogen peroxide with production of reactive oxygen species (ROS) can significantly inhibit the growth of subcutaneously implanted HeLa tumors in BALB/c mice. Tumor inhibition rates of 99% could be achieved when NPs were combined with H_2_O_2_ [149]. Furthermore, as shown by Kim et al., ultrasmall NPs could induce ferroptosis via the enhanced generation of ROS in cancer cells (Fig. 3) that was abrogated by the application of liproxstatin-1, an inhibitor of ferroptosis [150]. In another study, ferumoxytol, an FDA-approved iron supplement, induced polarization of tumor-associated macrophages into pro-inflammatory type 1 macrophages that corresponded with an inhibition of growth of subcutaneous adenocarcinomas in mice [151]. Taking into consideration the immunomodulatory and anti-tumor effects of MNPs, further combinations with other immunotherapeutic approaches, particularly with ICIs, might further improve melanoma theranostics (Fig. 3).

### Theranostic approaches based on extrinsic properties of nanoparticles

Apart from applications of MNPs as tools for direct tumor eradication due to their unique intrinsic characteristics, also other types of NPs are employed for a targeted melanoma theranostics in combination with other treatment modalities (Fig. 3). Their properties can be improved by introducing biocompatible and biodegradable coatings and by attaching various targeting and diagnostic therapeutic agents to their surface. It was demonstrated that decoration of particle surfaces with anti-melanoma agents could result in an enhanced potential in the delivery of chemotherapeutic drugs into tumor cells, thereby avoiding side effects. The most studied nanoparticle system, PEG-PLGA, mediates effective anti-melanoma effects [152–154]. In a recent work of Zhou et al., it was demonstrated that celastrol-containing PEG-PLGA NPs coated with membranes of neutrophils displayed significantly enhanced cytotoxicity and apoptosis rate in a B16F10 melanoma mouse model [155]. In another study, fabricated PLGA containing ursolic acid (UA) (pentacyclic triterpenoid extracted from plants) also demonstrated a therapeutic efficacy in the management of melanoma [156]. To further potentiate the therapeutic properties, these particles could be loaded with magnetic active substances such as iron. Liposomes loaded with dichloro(1,10-phenanthroline) copper (II) (CuPhen), a cytotoxic metallodrug, enabled iron oxide nanoparticles (IONPs) to retain their magnetic properties and to exert anti-melanoma effects [157]. Indeed, inclusion of iron oxide provides the possibility for an MR-guided assessment of tumor volume and allows the monitoring of therapy responses. MR imaging-guided chemotherapy by LDH-stabilized ultrasmall iron oxide Fe_3_O_4_ NPs coated with hyaluronic acid (HA) and loaded with the anticancer drug doxorubicin (DOX) demonstrated efficiency in melanoma treatment [158]. In addition to the loading with chemotherapeutic agents, magnetic NPs can also be conjugated to other molecules (e.g., fluorescent dyes, radionuclides, contrast-enhancing agents for MRI, siRNA, shRNA, etc.) to improve their theranostic capacity. A promising approach is the delivery of immunoadjuvants such as agonists for pattern recognition receptors by NPs [159]. Activation of TLR has been shown to modulate immune responses by stimulating recruitment and effector functions of T cells [21, 160–164]. In fact, several TLR ligands have already been coupled to NPs and used in preclinical models for cancer immunotherapy [165].

Accumulation of non-targeted magnetic particles in the tumor tissue occurs due to the enhanced permeability and retention effect (EPR). However, undesirable off-target uptake of NPs by the reticuloendothelial system (particularly in liver, spleen, and lungs) cannot be ruled out that can lead to toxic side effects. Inclusion of iron into NPs can enable magnetic targeting of NPs to the region of interest inside the body. Thus, cell-penetrating peptides (CPP) and TNF-α (CTNF-α)-anchored exosomes coupled to superparamagnetic iron oxide NPs (CTNF-α-exosome-SPIONs) showed an enhanced membrane targeting in a melanoma model when an external magnetic field was applied [143].

Among tumor-associated antigens for the development of targeted NPs, the 70 kDa heat shock protein Hsp70 is of particular interest as the presentation of this protein on the cell surface of tumor cells was shown in a large variety of solid tumors, hematological malignancies and melanoma but not on corresponding normal cells [166–168]. The tumor-specific cell surface localization of Hsp70 could be explained by an association of the chaperone with globotriaosylceramide Gb3, a tumor cell-specific sphingolipid residing in cholesterol-rich microdomains [169]. Subsequent in vitro studies clearly demonstrated that Hsp70 predominantly attaches to artificial liposomes that contain Gb3 (PC/SM/Chol/Gb3 at a ratio of 17/45/33/5), indicating that Gb3 is indeed an interacting partner of Hsp70 [169]. Apart from Gb3, phosphatidylserine (PS), a non-lipid raft component, also was shown to interact with Hsp70 in stressed tumor cells [170, 171]. Indeed, decoration of the nanoparticle surface with anti-Hsp70 bioligands (i.e., monoclonal antibodies, Fab-fragments of antibodies, peptides) significantly increased the targeting potential of the applied nanomaterials, thus enhancing their diagnostic properties in guided detection of the tumor employing magnetic resonance imaging (MRI), computed tomography (CT), positron emission tomography (PET) and fluorescent imaging [34, 172–175]. Furthermore, the attachment of therapeutic molecules to NPs targeting membrane-bound Hsp70 on tumor cells has the potential to further enhance anti-melanoma properties of MNPs. Granzyme B, a serine protease, has been shown to interact with membrane-bound Hsp70 on tumor cells [169]. Upon binding and uptake, granzyme B induces apoptosis selectively in tumor cells. Therefore, a decoration of MNPs with granzyme B resulted not only in an efficient homing of NPs to tumor cells, but also provides therapeutic effects via the stimulation of a granzyme B-mediated apoptosis ^34^.

## Conclusions

NPs and MNPs have been widely applied in the therapy of cancer. Due to their biophysical properties, they improve the accuracy of diagnosis and increase the efficacy of therapy. Over the last decade, the composition as well as the targeting properties (e.g., fluorescent dyes, radionuclides, chemotherapeutic molecules, antibodies, etc.) of MNPs has been optimized.

Another promising approach involves the combination of nanoparticle-based theranostics with other treatment modalities (i.e., radio- and/or chemotherapy, immunotherapy, targeted therapy, etc.) that can help to achieve a synergistic anti-melanoma effect. In conclusion, these further developments of nanoparticle composition through molecular tuning supported by comprehensive analysis could lead to the establishment of novel nanoplatforms for melanoma therapy (Fig. 3).

## Data Availability

Not applicable.

## Abbreviations

AJCC: American Joint Committee on Cancer
AMF: Alternating electromagnetic field
ICI: Immune checkpoint inhibitor
PD-1: Death protein 1
CPP: Cell-penetrating peptide
CT: Computed tomography
CM: Cutaneous melanoma
CTLA-4: Cytotoxic T-lymphocyte-associated protein 4
DOX: Doxorubicin
EPR: Enhanced permeability and retention effect
ERK: Extracellular signal-regulated kinase
Fab: Fragment antigen binding
FDA: Food and Drug Administration
Gb3: Globotriaosylceramide 3
Hsp: Heat shock protein
Lf: Low frequency
irAEs: Immune-related adverse events
IONP: Iron oxide nanoparticle
JNK: C-Jun-N-terminal kinase
MH: Magnetic hyperthermia
mLNV: Magnetic lipid nanocomposite vehicle
MNP: Magnetic nanoparticle
MRI: Magnetic resonance imaging
Tis: Tumor in situ
MAPK: Mitogen-activated protein kinase
nsPEF: Nanosecond pulsed electric field
PS: Phosphatidylserine
PHION: Porous hollow iron oxide nanoparticles
PET: Positron emission tomography
ROS: Reactive oxygen species
SPION: Superparamagnetic iron oxide nanoparticle
T-VEC: Talimogene laherparepvec
TCR: T cell receptor
TMB: 3,3',5,5'-Tetramethylbenzidine
TCGA: The Cancer Genome Atlas
TLR: Toll-like receptor
TNF: Tumor necrosis factor
TNM system: Tumor, node, metastasis system
UV: Ultraviolet
UA: Ursolic acid
VLP: Virus-like nanoparticle
